# Supplementary material for: ACEtimation—The Combined Effect of Adverse Childhood Experiences on Violence, Health-Harming Behaviors, and Mental Ill-Health: Findings across England and Wales
Source: Int J Environ Res Public Health. 2023 Aug 23;20(17):6633. doi: 10.3390/ijerph20176633 (PMC10487644; doi:10.3390/ijerph20176633)
Supplement: Supplementary file 1 [file ijerph-20-06633-s001.zip › ijerph-2520525-supplementary.pdf]

Supplementary S1

Table S1. Survey stratification, recruitment methods and sample sizes ……………………………………………pg2

Table S2. Derivation of predictor and outcome variables from questionnaires across surveys……………………......pg3

Table S3. Distribution of outcome responses across surveys………………………………………………………..pg6

Table S4a/b. Relative Risk Ratios for violence outcomes and substance use behaviors by demographic characteristics (RR [95% CIs], p values), Relative Risk Ratios for health harming behaviors and mental health outcomes by demographic characteristics (RR [95% CIs], p values)…….pg 7

Table S5. Estimated Marginal Means for all individual ACE exposures and combinations, including excess risk……pg 9

Supplementary S2 Methods for calculating additive risk (adapted methods from Anderson et al., 2005)…………………………………pg 12

Supplementary S1

Table S1. Survey stratification, recruitment methods and sample sizes

Study	Sampling stratification	Recruitment method	Participation rate	Total sample	Included sample
England 2012 (Northwest)	Deprivation quintile	Household survey	70.4%	1500	1421
England 2013	English Administrative Regions (n=10), deprivation quintile	Household survey	53.5%	4010	3885
England 2015 (South England)	Luton, Hertfordshire, Northamptonshire (n=3), deprivation/ ethnicity/urbanicity	Household survey	55.8%	5623	5454
Wales 2015	Welsh Health Regions (n=7), deprivation quintile	Household survey	49.1%	2028	2028
Wales 2017	Welsh Health Regions (n=7), deprivation quintile	Household survey	58.5%	2497	2497
England 2020-21 (North West)	Welsh Health Regions (n=7), deprivation quintile, age group	Telephone and online survey	30.8%	1895	1819
Wales 2020-21	Deprivation quintile, age group	Telephone and online survey	36.4%	2976	2872
England & Wales 2022	Country quotas representative by age and sex	Online survey	N/A	1832	1740
			Total	**22361**	**21716**

Table S2. Derivation of predictor and outcome variables from questionnaires across surveys

Variables	Questions used to derive response	Qualifying response
Child maltreatment	How often did a parent or adult in your home ever hit, beat, kick, or physically hurt you in any way? This does not include gentle smacking for punishment.<br><br>How often did a parent or adult in your home ever swear at you, insult you, or put you down?<br><br>Studies 1-5: How often did anyone at least 5 years older than you (including adults) ever….<br>…touch you sexually?<br>…try to make you touch them sexually?<br>…force you to have any type of sexual intercourse (oral, anal, or vaginal)?<br><br>OR<br><br>Studies 6-8: Did an adult or someone at least 5 years older than you sexually abuse you by touching you or making you undertake any sexual activity with them?	*Once or more than once to any of the questions*<br><br>*OR*<br><br>*Yes*
Witnessing violence	How often did your parents or adults in your home ever slap, hit, kick, punch, or beat each other up?	*Once; more than once*
Household dysfunction	Did you live with anyone who was depressed, mentally ill, or suicidal?<br><br>Did you live with anyone who was a problem drinker or alcoholic?<br><br>Did you live with anyone who used illegal street drugs or who abused prescription medications?<br><br>Did you live with anyone who served time or was sentenced to serve time in a prison or young offenders' institution?	*A yes response to any/all of these questions.*
Perpetrator of physical violence	Studies 1-3,5: How many times have you physically hit someone in the past 12 months?<br><br>Study 4: In the past 12 months, have you physically hit someone else?	*Once or more*<br><br>*Yes*

	Studies 6-7: In the last 12 months have you hit another adult for any reason, including to defend yourself?	*Yes*
Victim of physical violence	Studies 1-3,5: How many times have you been physically hit in the past 12 months?<br><br>Study 4: In the past 12 months, have you been physically hit by someone else?<br><br>Studies 6,7: In the last 12 months have you been hit by an adult?	*Once or more*<br><br><br>*Yes*<br><br><br>*Yes*
Incarceration	Studies 1-3,5: How many nights have you ever spent in prison, in jail or in a police station?<br><br>Study 4: Have you ever spent a night locked up in a prison, in jail or in a police station?	*Yes*
Binge drinking *(Current)*	Studies 1-3,5: How often do you have 6 or more standard drinks on one occasion?<br>Studies 6-8: Currently, on how many days a week do you drink five or more alcoholic drinks in one day (e.g., glasses of wine, beers, shots of spirits)?	*Weekly; Daily or almost Daily*<br><br><br>*1+ Days*
Cannabis use *(ever)*	Studies 1-5,8: How often, if ever, have you taken cannabis?<br><br>Studies 6,7: Please can you tell us if you have ever used the following and if you have used them in the last 12 months…Cannabis	*Used in the past 12 months; Used but not in the last 12 months; Used in the past month*
Smoking *(current)*	Studies 1-5: In terms of smoking (tobacco), which best describes you?<br><br>Studies 6,7: Please can you tell us if you have ever used the following and if you have used them in the last 12 months… Cigarettes<br><br>Study 8: In terms of smoking tobacco, which best describes you?	*I smoke daily; I smoke occasionally but not daily*<br><br>*I currently smoke tobacco*<br><br><br>*Used in the past 12 months*
Sexually transmitted infection	Studies 1-4, 6-8: Has a doctor or nurse ever told you that you have a sexually transmitted infection (e.g., chlamydia, HIV/AIDS, syphilis)	*Yes*
Underage sexual intercourse	Studies 1-4; How old were you the first time you had sexual intercourse?	*<16 years of age*

Accidental teenage pregnancy	Studies 1-4; Did you ever accidentally get pregnant or accidentally get someone else pregnant before you were aged 18 years?	*Yes once, yes more than once*
Life satisfaction	Studies 1-4; how satisfied are you with your life on a scale of 1-10?	*Score of 5 =≤*
Any mental illness diagnosis	Studies 6-8; Have you ever been told by doctor have mental illness, including depression and anxiety	*Yes*

Table S3. Distribution of outcome responses across surveys

**Study**	**Outcome variables**										
	Antisocial outcomes			***Risky health behaviours***						***Mental health outcomes***	
	Victim of physical violence	Perpetrator of physical violence	Incarceration	Binge drinking	Cannabis use	Smoking	Sexually transmitted infection	Early sexual initiation	Accidental teenage pregnancy	Low life satisfaction	Any Mental illness diagnosis
England 2012 (North West)	✓	✓	✓	✓	✓	✓	✓	✓	✓	✓	
England 2013	✓	✓	✓	✓	✓	✓	✓	✓	✓	✓	
England 2015 (South)	✓	✓	✓	✓	✓	✓	✓	✓	✓	✓	
Wales 2015	✓	✓	✓	✓	✓	✓	✓	✓	✓		
Wales 2017	✓	✓	✓		✓	✓					
England (North West) 2020-21	✓	✓		✓	✓	✓	✓				✓
Wales 2020-21	✓	✓		✓	✓	✓	✓				✓
England & Wales 2022				✓	✓	✓	✓				✓
Yes responses (n)	938	750	1112	2999	3843	4025	430	1888	894	1265	1743
No responses (n)	17947	18126	14140	16195	17788	11256	17624	8972	11788	9464	3550

Table S4a. Relative Risk Ratios for violence outcomes and substance use behaviors by demographic characteristics (RR [95% CIs], p values)

	Victim of violence	Perpetrator of violence	Incarcerated (ever)	Smoking	Binge drinking	Cannabis use (ever)
	**n= 18885**	**n= 18876**	**n= 15252**	**n= 21689**	**n= 19194**	**n= 21631**
Age						
60-69	0.1 [0.1, 1.1] **p <.001**	0.1 [0.1, 0.2] **p<.001**	0.6 [0.5, 0.7] **p<.001**	0.5 [0.4, 0.5] **p <.001**	0.5 [0.5, 0.6] **p <.001**	0.3 [0.2, 0.3] **p <.001**
50-59	0.2 [0.2, 0.3] **p <.001**	0.2 [0.2, 0.3] **p <.001**	1.0 [0.8, 1.1] p =.769	0.7 [0.7, 0.8] **p <.001**	0.9 [0.8, 1.0] **p = .004**	0.5 [.04, 0.5] **p <.001**
40-49	0.4 [0.3, 0.5] **p<.001**	0.4 [0.3, 0.5] **p<.001**	1.0 [0.9, 1.2] p = .682	0.8 [0.7, 0.9] **p <.001**	0.9 [0.8, 1.0] **p =.006**	0.7 [0.6, 0.8] **p <.001**
30-39	0.6 [0.5, 0.7] **p<.001**	0.6 [0.5, 0.7] **p<.001**	1.1 [1.0, 1.3] p =.173	0.9 [0.8, 1.0] **p = .040**	0.8 [0.7, 0.9] **p <.001**	0.9 [0.8,1.0] **p = .012**
18-29*	.	.	.	.	.	.
Gender						
Female	0.5 [0.4, 0.6] **p<.001**	[0.4, 0.6] **p<.001**	0.2 [0.2, 0.3] **p <.001**	0.8 [0.7, 0.8] **p <.001**	0.5 [0.5, 0.6] **p <.001**	0.6 [0.5, 0.6] **p <.001**
Male*	.	.	.	.	.	.
Ethnicity						
Other than white	0.8 [0.1, 0.9] **p = .005**	0.8 [0.7, 1.0] p=.104	0.5 [0.4, 0.7] **p<.001**	0.4 [0.4, 0.5] **p <.001**	0.3 [0.2, 0.4] **p <.001**	0.4 [0.4, 0.5] **p <.001**
White*	.	.	.	.	.	.
Deprivation Quintile						
5 Most deprived	1.2 (1.0, 1.5) p = .052	1.2 [1.0, 1.5] p = 0.69	2.3 [1.9, 2.7] **p <.001**	2.1 [1.9, 2.3] **p<.001**	0.9 [0.8, 1.0] p = .112	1.1 [1.0, 1.2] p = .184
4	1.2 [1.0, 1.5] p = .072	1.2 [1.0, 1.5] p = .083	1.8 [1.5, 2.2] **p < .001**	1.6 [1.4, 1.7] **p <.001**	0.7 [1.0, 0.9] p = .701	1.0 [0.9, 1.2] p = .456
3	1.1 [0.9, 1.4] p= .293	1.0 [0.8, 1.3] p = .873	1.5 [1.3, 1.9] p <.001	1.4 [1.2, 1.5] **p <.001**	1.0 [0.9, 1.1] p = .886	1.1 [1.0, 1.2] p =.313
2	1.1 [0.9, 1.4] p = .252	1.1 [0.9, 1.4] p = .297	1.2 [1.0, 1.5] p = .085	1.3 [1.1, 1.4] **p <.001**	1.0 [0.9, 1.1] p = .736	1.1 [1.0, 1.2] p = .375
1 Least deprived*	.	.	.	.	.	.

Note: *This denotes the variable was the reference category across analyses. Significant effects in bold.

Table S4b. Relative Risk Ratios for health harming behaviors and mental health outcomes by demographic characteristics (RR [95% CIs], p values)

	STI (ever)	Early sexual initiation	Accidental teenage	Low life satisfaction	Any mental illness
	**n= 18054**	**n= 10860**	**n= 12682**	**n= 10729**	**n= 5293**
Age					
60-69	0.4 [0.3, 0.6] **p <.001**	0.3 [0.2, 0.3] **p< .001**	0.6 [0.5, 0.8] **p < .001**	1.3 [1.1, 1.5] **p = .004**	0.9 [0.8, 1.0] **p = .034**
50-59	0.6 [0.4, 0.8] **p <.001**	0.5 [0.4, 0.6] **p <.001**	0.8 [0.7, 1.0] p = .067	1.8 [1.5, 2.1] **p <.001**	1.0 [0.9, 1.08] p = .462
40-49	0.7 [0.6, 1.0] **p =.028**	0.6 [0.5, 0.7] **p <.001**	0.9 [0.7, 1.0] p =.111	1.5 [1.3, 1.7] **p<.001**	1.1 [1.0, 1.3] p =.055
30-39	1.1 [0.8, 1.4] p = .586	0.7 [0.6, 0.8] **p <.001**	0.8 [0.7, 1.0] **p = .018**	1.1 [0.9, 1.3] p = .286	1.1 [1.0, 1.2] p = .084
18-29*	.	.	.	.	.
Gender					
Female	0.8 [0.6, 1.0] **p = .009**	0.7 [0.7, 0.8] **p <.001**	1.9 [1.6, 2.2] **p <.001**	0.9 [0.8, 1.0] p =.074	1.3 [1.2, 1.4] **p <.001**
Male*	.	.	.	.	.
Ethnicity					
Other than white	0.4 [0.3, 0.6] **p<.001**	0.4 [0.3, 0.4] **p <.001**	0.4 [0.3, 0.5] **p <.001**	0.8 [0.7, 1.0] p = .286	0.6 [0.5, 0.7] **p <.001**
White	.	.	.	.	.
Deprivation Quintile					
Most deprived	1.1 [0.8, 1.4] p = .677	1.5 [1.3, 1.7] **p <.001**	**1.8 [1.4, 2.2] p <.001**	2.1 [1.8, 2.5] **p <.001**	1.3 [1.2, 1.5] **p <.001**
4	1.0 [0.8, 1.4] p = .779	1.2 [1.05, 1.4] **p = .012**	1.5 [1.2, 1.8] **p <.001**	1.6 [1.4, 1.9] **p <.001**	1.2 [1.1, 1.4] **p = .003**
3	1.0 [0.7, 1.3] p =. 723	1.3 [1.2, 1.6] **p <.001**	1.7 [1.4, 2.1] **p <.001**	1.3 [1.1, 1.6] **p = .003**	1.1 [1.0, 1.3] p = .066
2	0.9 [0.7, 1.2] p =.460	1.1 [1.0, 1.3] p = .091	1.4 [1.2, 1.8] **p =.001**	1.05 [0.9, 1.3] p = .620	1.0 [0.9, 1.2] p = .650
1 Least deprived*	.	.	.	.	.

Note: *This denotes the variable was the reference category across analyses. Significant effects in bold.

Table S5. Estimated Marginal Means for all individual ACE exposures and combinations, including excess risk

ACE Exposure	Estimated Marginal Means (%)	ACE categories summed (%) *	Excess risk (%) ƚ
VICTIM OF VIOLENCE			
No ACEs	1.8%	.	.
Household dysfunction only	3.2%	.	.
Witnessed violence only	2.9%	.	.
Child maltreatment only	3.8%	.	.
Household dysfunction and witnessed violence	6.6%	2.5%	2.4%
Child maltreatment and household dysfunction	7.2%	3.4%	2.0%
Child maltreatment and witnessed violence	7.0%	3.1%	2.2%
All three ACEs	10.2%	4.5%	3.9%
PERPETRATOR OF VIOLENCE			
No ACEs	1.2%	.	.
Household dysfunction only	2.9%	.	.
Witnessed violence only	1.3%	.	.
Child maltreatment only	3.4%	.	.
Household dysfunction and witnessed violence	5.6%	1.7%	2.6%
Child maltreatment and household dysfunction	6.0%	3.8%	1.0%
Child maltreatment and witnessed violence	5.1%	2.2%	1.7%
All three ACEs	8.9%	3.9%	3.8%
INCARCERATION			
No ACEs	2.3%	.	.
Household dysfunction only	5.3%	.	.
Witnessed violence only	4.7%	.	.
Child maltreatment only	4.9%	.	.
Household dysfunction and witnessed violence	8.0%	5.4%	0.3%
Child maltreatment and household dysfunction	8.1%	5.6%	0.2%
Child maltreatment and witnessed violence	7.7%	4.9%	0.5%
All three ACEs	13.1%	7.9%	2.9%
BINGE DRINKING			
No ACEs	8.5%	.	.
Household dysfunction only	10.3%	.	.
Witnessed violence only	10.2%	.	.
Child maltreatment only	10.5%	.	.
Household dysfunction and witnessed violence	11.9%	3.5%	-0.1%
Child maltreatment and household dysfunction	10.6%	3.8%	-1.8%
Child maltreatment and witnessed violence	11.2%	3.7%	-1.0%
All three ACEs	14.8%	5.5%	0.7%
CANNABIS USE (EVER)			
No ACEs	7.7%	.	.
Household dysfunction only	13.2%	.	.
Witnessed violence only	10.9%	.	.
Child maltreatment only	13.8%	.	.

Household dysfunction and witnessed violence	15.6%	8.8%	-0.9%
Child maltreatment and household dysfunction	19.5%	11.7%	0.1%
Child maltreatment and witnessed violence	14.1%	9.4%	-2.9%
All three ACEs	21.9%	-0.7%	14.9%
EARLY SEXUAL INTITATION			
No ACEs	8.0%	.	.
Household dysfunction only	12.8%	.	.
Witnessed violence only	8.6%	.	.
Child maltreatment only	13.2%	.	.
Household dysfunction and witnessed violence	15.0%	5.4%	1.6%
Child maltreatment and household dysfunction	17.3%	10.1%	-0.8%
Child maltreatment and witnessed violence	14.9%	5.8%	1.1%
All three ACEs	20.4%	10.7%	1.7%
SEXUALLY TRANSMITTED INFECTION (EVER)			
No ACEs	0.9%	.	.
Household dysfunction only	1.9%	.	.
Witnessed violence only	1.0%	.	.
Child maltreatment only	1.8%	.	.
Household dysfunction and witnessed violence	2.5%	1.2%	0.5%
Child maltreatment and household dysfunction	2.5%	1.9%	-0.3%
Child maltreatment and witnessed violence	2.9%	1.1%	0.9%
All three ACEs	3.6%	2.1%	0.6%
ACCIDENTAL TEENAGE PREGNANCY			
No ACEs	3.2%	.	.
Household dysfunction only	5.1%	.	.
Witnessed violence only	4.4%	.	.
Child maltreatment only	5.6%	.	.
Household dysfunction and witnessed violence	5.4%	3.2%	-1.0%
Child maltreatment and household dysfunction	8.3%	4.4%	0.8%
Child maltreatment and witnessed violence	9.0%	3.6%	2.2%
All three ACEs	12.4%	5.6%	3.6%
LOW LIFE SATISFACTION			
No ACEs	7.5%	.	.
Household dysfunction only	12.1%	.	.
Witnessed violence only	10.0%	.	.
Child maltreatment only	13.5%	.	.
Household dysfunction and witnessed violence	14.6%	7.2%	0.0%
Child maltreatment and household dysfunction	22.4%	10.6%	4.3%
Child maltreatment and witnessed violence	12.6%	8.5%	-3.4%
All three ACEs	23.7%	13.2%	3.0%
ANY MENTAL HEALTH DIAGNOSIS (EVER)			
No ACEs	17.9%	.	.
Household dysfunction only	30.6%	.	.
Witnessed violence only	21.5%	.	.
Child maltreatment only	27.9%	.	.

Household dysfunction and witnessed violence	21.2%	16.4%	-13.1%
Child maltreatment and household dysfunction	37.8%	22.7%	-2.8%
Child maltreatment and witnessed violence	28.1%	13.7%	-3.5%
All three ACEs	36.7%	26.4%	-7.6%
SMOKING			
No ACEs	12.2%	.	.
Household dysfunction only	15.1%	.	.
Witnessed violence only	12.7%	.	.
Child maltreatment only	15.6%	.	.
Household dysfunction and witnessed violence	18.0%	3.4%	2.4%
Child maltreatment and household dysfunction	19.3%	6.3%	0.8%
Child maltreatment and witnessed violence	17.7%	3.9%	1.7%
All three ACEs	24.0%	6.8%	5.0%

Note: *This denotes the percentage of risk of each individual ACE category added to the other whilst subtracting the baseline effects (e.g., No ACEs). ł This denotes the excess risk over and above what would be expected when two or more ACE categories are combined.

Supplementary S2

Methods for calculating additive risk (adapted methods from Andersson et al., 2005)

1. Estimated Marginal Means for each level of the predictor (000, 001, 011, 010, 110, 100, 101, 111) were entered into excel and converted into percentages.
2. The baseline risk (000) was subtracted from the raw percentages of each level of the predictor. This was to ensure estimates accounted for baseline effects.
3. Then, the percentages from individual ACE categories were summed using percentages from step 2 to give an *expected value* of all two way and three-way ACE category combinations (i.e., 001 + 010 = 011, 100 +010 = 110, 001 + 100 = 101, 001+101 +010 = 111). This accounted for baseline effects.
4. Then, the *expected* values from step 3 (e.g., 001 + 010 = 011) were subtracted from the *observed* percentages of risk for all two way and three-way ACE category combinations (011, 110, 101, 111). This gave us a percentage of *excess risk.* That is, the percentage of risk observed when two or more ACE categories co-occur over and above what would be expected by summing their individual effects.

**Excel calculator template can be provided upon request*
